# Online prevention for patients with lifestyle related diagnoses

**Published:** 2012-06-15

**Authors:** Siv Söderberg, Inger Lindberg, Lennart Isaksson

**Affiliations:** eHealth Innovation Centre, Department of Health Science, Luleå University of Technology, Sweden; eHealth Innovation Centre, Department of Health Science, Luleå University of Technology, Sweden; eHealth Innovation Centre, Department of Health Science, Luleå University of Technology, Sweden

**Keywords:** prevention, lifestyle, telemonitoring, coaching, empowerment

## Abstract

**Aims and objectives:**

To evaluate whether the introduction of large-scale personalized and technology supported telemonitoring and health coaching interventions produces benefits in terms of health related quality of life, health status and empowerment of patients with a type-2 diabetes and cardiovascular disease (CVD).

**Introduction:**

People with lifestyle related diagnoses as type-2 diabetes and CVD are increasing. The Swedish field trial in the Renewing Health project have developed a method that combine health coaching with online-management of patients data where the patient becomes more actively involved in their own health and healthcare.

**Methods:**

The method is applied at field trials in four Primary healthcare centres in the county of Norrbotten, Sweden. The evaluation is made through a randomized trial with 700 patients with type-2 diabetes or CVD. Fifty percent of the participants take part in the intervention group. General practitioners, diabetic nurses, physiotherapists and nutritionists co-operate to manage the patient interactions. The technical implementation is based on a national patient portal. It provides secure access for all Swedish citizens to their health information and supports also electronic interaction with healthcare professionals. During the field trial, 85% of the patients use their own computers, the others are provided with Tablet-PCs. The preventive health and diagnose measurement equipment are also provided to the patients for free during the field trial. But for large scale usage, the intention is that most of the equipment will be owned by the patients. The patients have attended group sessions were they were educated and motivated to perform lifestyle changes. They were trained on how to manage their health information and interact with the health professionals. Individualized health activity plans were developed. The patients perform health promotion activities and report parameters like number of steps, pulse and duration. They also measure and report medical parameters like blood pressure, blood glucose, PK values and 2-channel ECG. The health professionals provide reference values that make it intuitively for the patient to interpret the progress through graphical diagrams. About every 2nd month, the results are reviewed and the healthcare activity plan is revised if needed. When applicable, alarm levels are specified. Since improved lifestyle can reduce the need for medical treatment, the alarms make the healthcare professionals aware of the necessity to change prescription. The patients have on-line access to actual medication list and can select to have a notification when it is time to take medication. They can also report and follow-up performed medication.

**Results:**

The health coaching methodology has been refined for larger scale usage. The new functionality has been integrated with the Swedish national patient portal. That makes it possible for all Swedish healthcare organizations to offer the Prescribed Healthcare method to their patients after the field trial. The field trial evaluation will be completed Q4-2013.

**Conclusions:**

By combining health coaching services with widely available functionality for treatment instructions, preventive health- and medical diagnose measurement, visualization and medication support engage and stimulate patients to adopt a healthier lifestyle and improves compliance to medication and the activity plan.

